# Chronic diseases and productivity loss among middle-aged and elderly in India

**DOI:** 10.1186/s12889-022-14813-2

**Published:** 2022-12-16

**Authors:** Shamrin Akhtar, Sanjay K. Mohanty, Rajeev Ranjan Singh, Soumendu Sen

**Affiliations:** 1 International Institute for Population Sciences, Mumbai, Maharashtra 400088 India; 2 Department of Population and Development, International Institute for Population Sciences, Mumbai, 400088 India

**Keywords:** Chronic diseases, Ever-stopped work, Limiting paid work, Elderly, Middle-aged, Productivity loss, India

## Abstract

**Context:**

Chronic diseases are growing in India and largely affecting the middle-aged and elderly population; many of them are in working age. Though a large number of studies estimated the out-of-pocket payment and financial catastrophe due to this condition, there are no nationally representative studies on productivity loss due to health problems. This paper examined the pattern and prevalence of productivity loss, due to chronic diseases among middle-aged and elderly in India.

**Methods:**

We have used a total of 72,250 respondents from the first wave of Longitudinal Ageing Study in India (LASI), conducted in 2017-18. We have used two dependent variables, limiting paid work and ever stopped work due to ill health. We have estimated the age-sex adjusted prevalence of ever stopped working due to ill health and limiting paid work across MPCE quintile and socio- demographic characteristics. Propensity Score Matching (PSM) and logistic regression was used to examine the effect of chronic diseases on both these variables.

**Findings:**

We estimated that among middle aged adults in 45–64 years, 3,213 individuals accounting to 6.9% (95%CI:6.46–7.24) had ever-stopped work and 6,300 individuals accounting to 22.7% (95% CI: 21.49–23.95) had limiting paid work in India. The proportion of ever-stopped and limiting work due to health problem increased significantly with age and the number of chronic diseases. Limiting paid work is higher among females (25.1%), and in urban areas (24%) whereas ever-stopped is lower among female (5.7%) (95% CI:5.16–6.25 ) and in urban areas (4.9%) (95% CI: 4.20–5.69). The study also found that stroke (21.1%) and neurological or psychiatric problems (18%) were significantly associated with both ever stopped work and limiting paid work. PSM model shows that, those with chronic diseases are 4% and 11% more likely to stop and limit their work respectively. Regression model reveals that more than one chronic conditions had a consistent and significant positive impact on stopping work for over a year (increasing productivity loss) across all three models.

**Conclusion:**

Individuals having any chronic disease has higher likelihood of ever stopped work and limiting paid work. Promoting awareness, screening and treatment at workplace is recommended to reduce adverse consequences of chronic disease in India.

**Supplementary Information:**

The online version contains supplementary material available at 10.1186/s12889-022-14813-2.

## Introduction

Ill-health, work, and productivity are interrelated. The pro-longed ill-health due to chronic diseases has a higher chance of premature mortality [1], increasing the chance of disability [2], higher use of medical services and exerts greater economic burden to household and nation. At the households level, economic burden can be both direct and indirect [3]. The high out-of-pocket spending, catastrophic health spending and impoverishment are direct consequences of increasing chronic diseases [4]. Indirect burden of chronic diseases includes work absenteeism, voluntary retirement from work [5], and reduced propensity to work [6]. The cascading effect of ill-health reduces individual income [7] and may lead to poor physical and mental health [8] and may lead to gradual loss of productivity and welfare.

Productivity loss reduces the income and well-being of individuals and households. Ill-health often reduces the work participation as it affects the prime working age group. Productive time forgone due to ill-health cost both, to the household and the nation as well. Productivity loss is measured using multiple indicators; work absenteeism, presenteeism, permanent withdrawal from the workforce, and job interruption [9]. While work absenteeism refers to absence due to illness, presenteeism is low work performance during sickness [10]. Permanent withdrawal from the workforce includes voluntary retirement due to impairment or other health problems. Work-related injuries or accidents and success and failure also add to productivity loss [11].

Most of the studies on the consequences of chronic diseases on work productivity were carried out in developed countries [12–14]. People with poor health are more likely to spend a considerable time in seeking healthcare and that may lead to work absenteeism [15]. Among respondents who experienced symptoms related to health conditions in Germany, the average number of workdays lost due to absenteeism and presenteeism was 27 days per respondent annually [16]. Results from a study in Australia shows that the full-time workers with mental disorders lost an average of one day due to absenteeism and three days due to presenteeism in one month reference period [17]. In USA, the weekly absenteeism costs US$1685/employee per year and about 71% of the total productivity loss was contributed by reduced performance at work [18]. Asthma, cancer, heart disease, and respiratory disorders were estimated to have presenteeism costs of more than US$200 per person annually in USA [19]. Presenteeism represents the largest component and leading driver to the medical costs, specifically among the patients with migraine/headache, allergies, and arthritis [20]. Depression ranked third among health conditions with an annual productivity loss of US$878 per person [21]. A higher number of health risks is associated with lower on-the-job productivity [22]. Adults with multiple chronic diseases may have high chance of reduced productivity [23] In India, nearly a quarter of the companies lose approximately 14% of the total working days annually due to sickness [24].

**Fig. 1:**
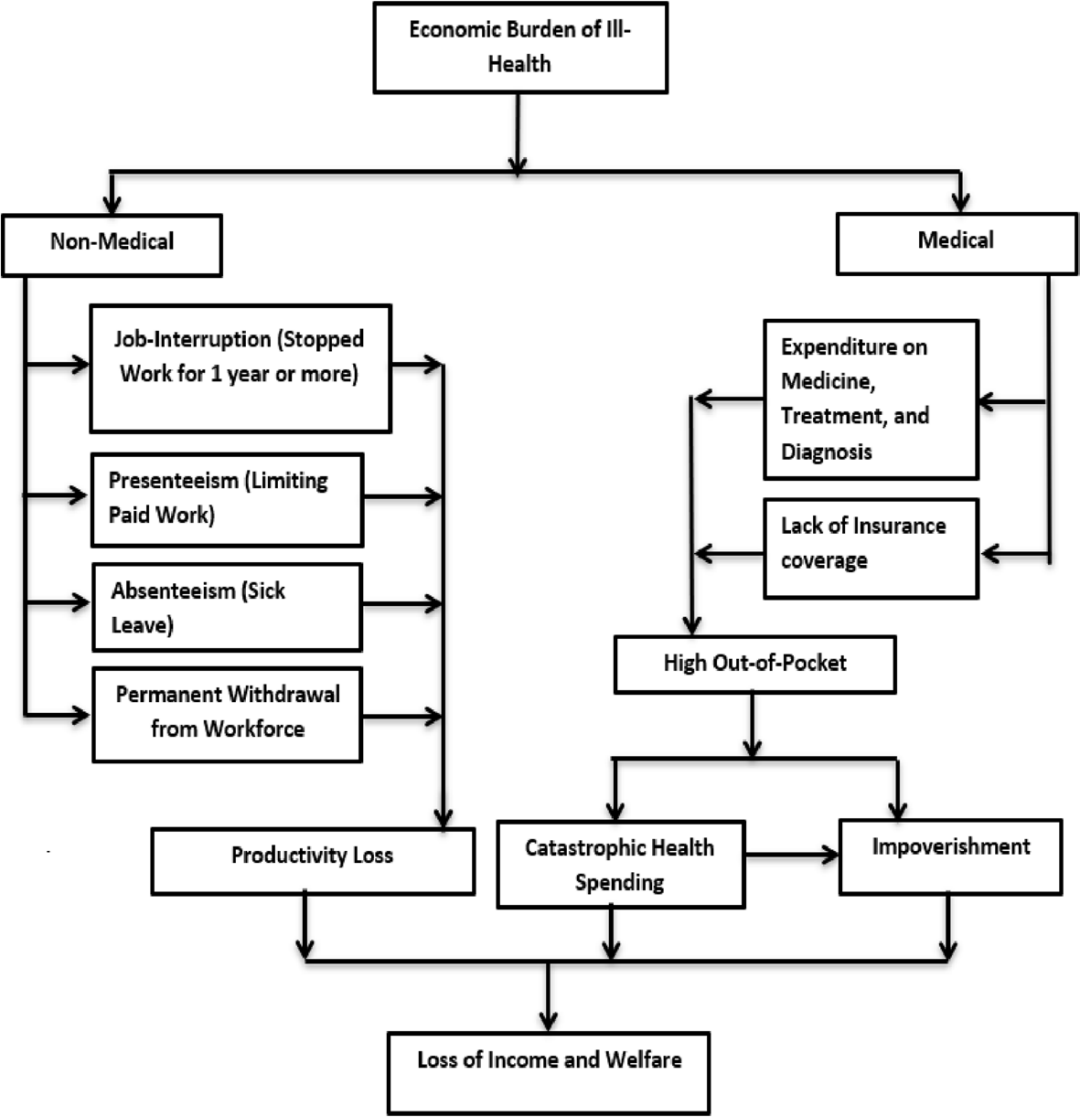
A framework on economic burden of ill-health

Older adults in India are vulnerable to chronic diseases and, that may affect their work temporary or permanently [25]. The country has achieved the replacement level of fertility and nearing completion of demographic transition, resulting increasing share of older adults and elderly in the country and increasing burden of non-communicable disease (NCD). The share of middle aged and elderly population (45+) has increased from 18.9% to 2001 to 25.1% by 2020 [26]. The median age of onset of NCDs was also declining from 57 years in 2004 to 53 years by 2018 [27]. Though large number of studies estimated the OOP and catastrophic health spending, socio-economic inequality and determinant of OOPS and CHE [28], there is no nationally representative studies on productivity loss due to health problems. Present study explores the pattern and prevalence of limiting paid work and productivity loss among middle-aged and elderly in India and their association with chronic diseases. Figure 1 presents a schematic presentation of productivity loss. It depicts the pathways how economic burden of ill-health lead to loss of income and welfare through various medical and non-medical components. The non-medical component includes absenteeism, presenteeism and job-interruption.

### Data and methods

#### Data

The study utilizes data from the first wave of Longitudinal Ageing Study in India (LASI), collected during April 2017 to December 2018. The survey was conducted by International Institute for Population Sciences (IIPS) in collaboration with Harvard T.H. Chan School of Public Health (HSPH), University of Southern California (USC) and other national institutions. Using multistage sampling method, a total of 42,949 households and 72,250 individuals aged 45 years and older and their spouses were successfully interviewed. Among these individuals, a total of 3,213 ever stopped working for a year or more due to health problem and 6,300 had limiting paid work. The data is publicly available for all states except Sikkim at the time of drafting this paper. The household and individual response rate was 95.8% and 87.3% respectively. Detailed about the survey and the findings are available in national report [29].

### Variable description

#### Outcome variables

In LASI survey, a detailed module on ever work, current work, stopped work and limiting paid work due to health issues were collected. The questions on stopped work begins with *“have you ever stopped working for one year or more at a time due to reasons of family, health, education, economic recession, natural disasters, etc.?”* and the question on limiting work reads as *“Do you have any impairment or health problem that limits the kind or amount of paid work you can do?”.* We used ever stopped work (1 = yes, 0 = no) for one year or more due to health problem and whether health problem had limit the paid work (1 = yes, 0 = no) as two outcome variables.

#### Covariates

We have used a set of demographic, economic, behavioural and health covariates in the analyses. These includes age (45–54, 55–64, 65–74, 75+), sex (male/female), educational attainment (illiterate, less than 5 years, 5–9 years completed, 10 years or more), monthly per capita expenditure quintile (MPCE), place of residence (rural/urban), caste (scheduled caste, scheduled tribe, other backward classes, others), religion (Hindu, Muslim, Christian, others), marital status (currently married, widowed, others) and regions (north, central, east, northeast, west, south) were used as the predictors in this study. The MPCE was used to depict the living standard of the household. In addition, the number of chronic diseases (hypertension, diabetes, chronic lung disease, chronic heart diseases, stroke, arthritis, neurological or psychiatric problems), health insurance coverage (yes/no), practicing exercise (yes/rarely/never) and smoking tobacco (yes/no) are included to examine their association with the limiting paid work or ever stopping work for one year or more among older adults.

#### Treatment variable for PSM

In LASI, respondents were asked if they were diagnosed with chronic disease such as hypertension, diabetes, cancer, chronic lung disease, chronic heart disease, stroke, arthritis, and neurological problem. The individuals who had reported being diagnosed with any chronic diseases (1 = yes, 0 = no) have been considered as treatment group and those not being reported any of the chronic diseases have been treated as control group in the study. The treatment and control group did not overlap as they were mutually exclusive in nature.

### Statistical analysis

Descriptive statistics, age-sex adjusted estimates, propensity score matching and logistic regression model were used in the analysis.

#### Prevalence of ever stopped work and limiting paid work

We estimated age-sex adjusted prevalence of ever stopped working and limiting paid work using the nationally representative full sample age-sex composition as reference using logistic regression.

#### Propensity score matching analysis

The propensity score matching (PSM) considers the potential selectivity in the sample. PSM is a statistical technique that estimates the effect of an intervention or a treatment by adjusting for covariates that predicts the results of receiving the treatment [30]. The advantage of using PSM model is that it compares the treated and controlled group on the basis of similar observed characteristics [31, 32]. The PSM has been used for evaluating various programme in a number of research studies [31–34]. For determining the average treatment effect (i.e., the effect of having any chronic disease), a counterfactual model is estimated.

#### Propensity score

The PSM is the probability of the middle aged and elderly population who had chronic diseases with certain characteristics, may be written as,

1 $$\mathrm P(\mathrm X)\:=\:\Pr\;(\mathrm D\:=\:1\vert\;\mathrm X)$$

Where, D = 1 if the population had any chronic diseases D = 0, otherwise.

And X is the vector of all the covariates used in the model.

Generally, PSM model estimated three probabilities, such as, Average Treatment Effect on the Treated (ATT), Average Treatment Effect on the Untreated (ATU) and Average Treatment Effect (ATE).

ATE is the average treatment effect of the intervention variable on the outcome variable and can be explained by using following equation

2 $$\mathrm{ATE}\;=\;\mathrm E\;(\mathrm\delta)\;=\;\mathrm E\;({\mathrm Y}_1-{\mathrm Y}_0)$$

where E (.) means average and Y_1_ represents potential outcome for those having any chronic disease and Y_0_ represents potential outcome for the population having no chronic diseases.

With the help of counterfactual model, the ATT can be written as

3 $$\mathrm{ATT}\;=\;\mathrm E\;({\mathrm Y}_1/\mathrm D=1)-\mathrm E\;({\mathrm Y}_0/\mathrm D=1)$$

The counterfactual model is the potential outcome that would have been obtained in case of not having any chronic disease and vice versa.

Where, E (Y_1_/D = 1) is stopping work who have any chronic disease.

E (Y_0_/D = 1) is the expected outcome for the individuals having any chronic disease if they would not have any of the diseases.

Similarly, the average treatment effect on the untreated (ATU) is defined as:

4 $$\mathrm{ATU}\;=\;\mathrm E\;({\mathrm Y}_1/\mathrm D=\;0)\;-\;\mathrm E\;({\mathrm Y}_0/\mathrm D=0)$$

Where E (Y_1_/D = 0) is the expected outcome if the individuals without any chronic disease were to have any chronic disease.

E (Y_0_/D = 0) is the counterfactual model predicts the outcome for the individuals who would have had any chronic disease but earlier they had not any.

The average treatment effect (ATE) is the difference between the expected outcome for those with any chronic disease and those without any chronic disease.

We used psmatch2 command in the STATA 16 which provides all the estimates using Mahalanobis matching technique.

#### Logistic regression

We used the multivariate logistic regression as a robustness check in support to our PSM model. We used three different models to understand the impact of each covariate on ever stopping work and limiting paid work separately. In the Model 1, we adjusted only for the number of chronic diseases. In model 2, socio-demographic variables were considered (age, sex residence, caste, religion, marital status and region). Finally, the socioeconomic variables along with smoking/substance abuse, exercise, health insurance and other predictors were adjusted in Model 3 to assess the adjusted effect of all the covariates on ever stopping work for one year or more. The following regression equation has been used.

$$\mathrm{Logit}\;({\mathrm Y}_{\mathrm i})\:=\:\ln(\mathrm p/1-\mathrm p)\;=\;\mathrm\alpha\:+{\:{\mathrm\beta}_{\mathrm i}\mathrm X}_{\mathrm i}$$

Where, Y is the probability of outcome event of the i^th^ individual. The model estimates the log odds of ever stopped work and limiting paid work adjusted for a set of explanatory variables (X_i_).

STATA version 16 was used for cleaning, standardizing data (to adjusted form), and for analysing data. Independent variables included individual level variables.

## Results

**Fig. 2:**
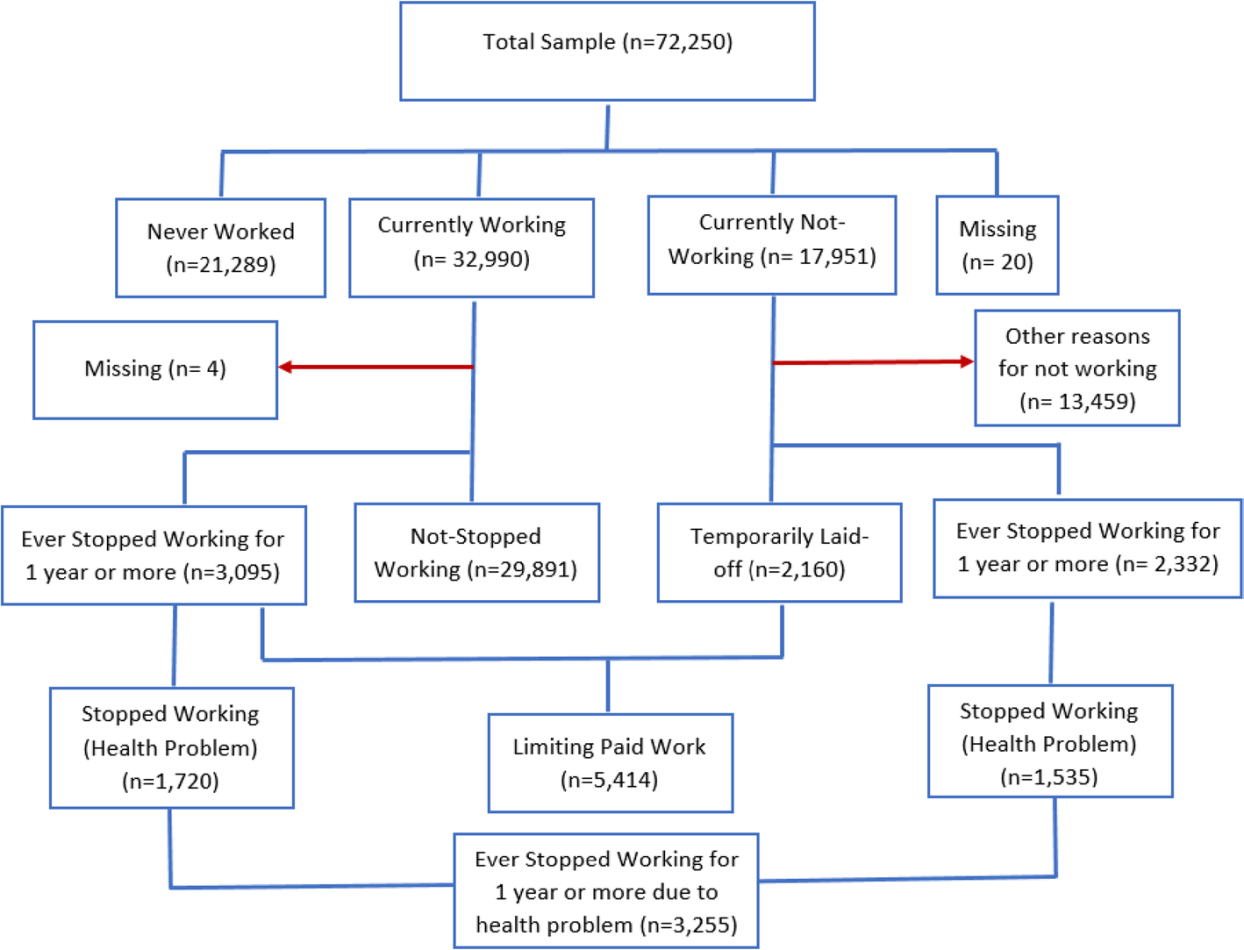
Schematic presentation of ever worked never worked, stopped and limiting work among middle age and elderly in India, 2017-18

Figure 2 shows a flow chart of participant selection for our analysis. Among 72,250 participants interviewed in LASI, 50,941 (72.4%) have ever worked and 21,289 (27.6%) had never worked. Those ever worked, 32,990 were currently working and 17,951 were not working currently. Those who were not working currently, about 31.5% have had stopped work, out of which health related reason accounts 56.5% followed by 20% due to childcare.

Table 1 presents the socio-economic and demographic profile of the study samples of ever worked and currently working/ temporarily laid off. Of the total surveyed individuals, 59.3% had ever worked and 40.7% were currently working/temporarily laid off. Over 67.52% of ever worked sample population were in the working age group (under 65) compared to 81.03% for currently working sample. The sample was predominantly rural and currently married. About 56.99% of ever worked sample did not had any chronic disease compared to 62.75% among currently working/ temporarily laid off. Majority of the respondents were illiterates. Sample were proportionately distributed across regions.

**Table 1:** Descriptive statistics of sample profile by socioeconomic and demographic characteristics among middle aged and elderly in India, 2017–18

	Had any chronic condition				Had no chronic condition			
	**Ever stopped work for a year or more due to health problem**		**Limiting paid work**		**Ever stopped work for a year or more due to health problem**		**Limiting paid work**	
	***N* = 1,660**		***N* = 2,973**		***N* = 1,553**		***N* = 3,327**	
	**Number**	**Percent**	**Number**	**Percent**	**Number**	**Percent**	**Number**	**Percent**
**MPCE Quintile**								
Poorest	331	19.94	570	19.17	401	25.82	849	25.52
Poorer	370	22.29	602	20.25	354	22.79	746	22.42
Middle	355	21.39	648	21.8	287	18.48	614	18.46
Richer	309	18.61	629	21.16	271	17.45	613	18.43
Richest	295	17.77	524	17.63	240	15.45	505	15.18
**Educational attainment**								
Illiterate	788	46.85	1,432	47.48	771	49.01	1,727	51
Less than 5 years	249	14.8	423	14.03	241	15.32	457	13.5
5–9 years completed	425	25.27	726	24.07	374	23.78	780	23.04
10 years or more	220	13.08	435	14.42	187	11.89	422	12.46
**Age**								
< 45	54	3.21	143	4.74	83	5.28	258	7.62
45–54	495	29.43	936	31.03	574	36.49	1,325	39.13
55–64	492	29.25	985	32.66	475	30.2	1,013	29.92
65–74	437	25.98	745	24.7	318	20.22	650	19.2
75+	204	12.13	207	6.86	123	7.82	140	4.13
**Sex**								
Male	1,090	64.8	1,800	59.68	997	63.38	1,988	58.71
Female	592	35.2	1,216	40.32	576	36.62	1,398	41.29
**Residence**								
Rural	1,226	72.89	2,118	70.23	1,210	76.92	2,624	77.5
Urban	456	27.11	898	29.77	363	23.08	762	22.5
**Caste**								
Scheduled Tribes	202	12.03	386	12.82	319	20.29	731	21.6
Scheduled Castes	343	20.43	619	20.56	345	21.95	666	19.68
OBC	754	44.91	1,318	43.79	596	37.91	1,316	38.89
Others	380	22.63	687	22.82	321	19.85	671	19.83
**Religion**								
Hindu	1,289	76.63	2,329	77.22	1,231	78.26	2,753	81.31
Muslim	195	11.59	383	12.7	154	9.79	277	8.18
Christian	123	7.31	164	5.44	120	7.63	177	5.23
Others	75	4.46	140	4.64	68	4.32	179	5.29
**Marital Status**								
Currently married	1,320	78.52	2,418	80.17	1,249	79.4	2,791	82.43
Widowed	298	17.73	513	17.01	269	17.1	502	14.83
Others	63	3.75	85	2.82	55	3.5	93	2.75
**Smoke/Substance use**								
Yes	848	50.69	1,395	46.59	836	53.9	1,615	48.05
No	825	49.31	1,599	53.41	715	46.1	1,746	51.95
**Practicing Exercise**								
Yes	158	9.5	296	9.98	114	7.38	260	7.78
Rarely/ Never	1,506	90.5	2,671	90.02	1,431	92.62	3,081	92.22
**Health Insurance**								
No	1,217	72.74	2,203	73.68	1,119	72.15	2,460	73.21
Yes	456	27.26	787	26.32	432	27.85	900	26.79
**Regions**								
North	293	17.42	508	16.84	199	12.65	445	13.14
Central	187	11.12	356	11.8	275	17.48	538	15.89
East	286	17	502	16.64	285	18.05	626	18.49
Northeast	135	8.03	108	3.58	182	11.57	102	3.01
West	293	17.42	775	25.7	286	18.18	1,056	31.19
South	488	29.01	767	25.43	347	22.06	619	18.28

**Fig. 3:**
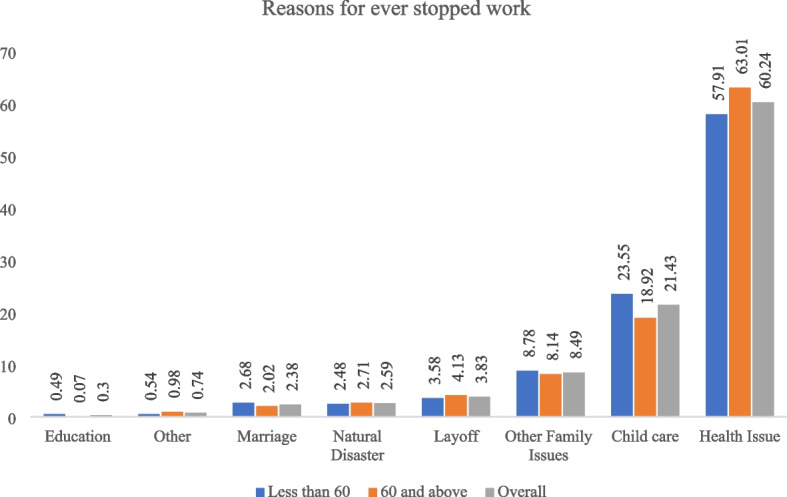
Percent distribution of middle-aged adults and elderly ever stopped work by reasons in India, 2017-18

Figure 3 shows reasons for ever stopped work among elderly and non-elderly in India. Health issue (60%) is the major reason for ever stopped work followed by child care (21%) and other family issues (9%). It is slightly higher for the elderly as compared to the middle-aged people. In case of child care, it is higher for the middle-aged people than elderly.

Table 2 presents the age-sex adjusted estimates of ever stopped work and limiting work (whose paid work was limited due to health reasons) by socioeconomic and demographic characteristics among individuals with and without chronic conditions. We estimated that 8.4% [95% CI: 7.52–9.24] older adults in India ever stopped work with a chronic condition compared to 5.35% [95% CI: 4.82–5.96] without chronic condition. Similarly, 31.1% [95% CI: 27.86–34.39] had limiting paid work compared to 18.3% [95% CI: 16.78–19.86] without any chronic condition. The proportion of ever stopped work for one year or more increases with age and decline with the level of education for both the group. The prevalence of stopped work among the treatment group was higher in urban areas (9.8%,95% CI: 9.04–10.54), among males (9.9%, 95% CI: 9.03–10.77) and among those who smoke/use any substance. However, no difference in prevalence were observed across different caste, religion and marital status in both treatment and control group. Notably, the prevalence of ever stopped work for one year or more was highest in poorest MPCE quintile (9.2%, 95% CI: 7.80-10.64) and lowest in richest MPCE quintile (6.7%, 95% CI: 5.33–8.07). However, the prevalence of ever stopped work and limiting paid work varied across the regions of India with highest being in western region in both the groups. The proportion of participants whose paid work was limited due to health reasons also increases with age and higher among females. It was higher in urban areas, and among those who smoke/use any substance. The prevalence of limiting paid work was higher among richest MPCE quintile compared to poorest MPCE quintile. Overall, for each of the background characteristics, prevalence was higher among the ones limiting paid work than those who ever stopped work for 1 year or more due to health reasons in both the groups in India. However, the prevalence of both the outcome variables were higher in the treatment group compared to that in the control group.

**Table 2:** Age-sex adjusted estimates of ever stopped work for one year or more and limiting paid work by socioeconomic and demographic characteristics among middle aged and elderly in India, LASI 2017–18

	Had any chronic condition		Had no chronic condition	
	***N* = 1,660**	***N* = 2,973**	***N* = 1,553**	***N* = 3,327**
	**Stopped work for 1 year or more due to health problem**	**Limiting paid work**	**Stopped work for 1 year or more due to health problem**	**Limiting paid work**
	**Prevalence (95% CI)**	**Prevalence (95% CI)**	**Prevalence (95% CI)**	**Prevalence (95% CI)**
**India**	**8.4 [7.52, 9.24]**	**31.1[27.86,34.39]**	**5.35 [4.82, 5.96]**	**18.3 [16.78, 19.86]**
**Age**				
< 45	8.7 [5.60, 11.87]	23.7 [17.93, 29.43]	4.5 [2.72, 6.36]	17.9 [8.93, 26.94]
45–54	8.8 [7.49, 10.01]	26.7 [20.98, 32.43]	5.2 [4.52, 5.83]	14.9 [13.73, 16.12]
55–64	8.6 [7.54, 9.74]	29.6 [26.98, 32.26]	5.9 [5.19, 6.64]	17.9 [16.57, 19.227]
65–74	8.5 [7.39, 9.54]	38.9 [35.84, 41.93]	5.8 [4.93, 6.59]	26.1 [23.54, 28.67]
75+	8.1 [6.36, 9.76]	44.0 [37.56, 50.50]	5.6 [4.14, 7.00]	31.5 [20.70, 42.20]
**Sex**				
Male	9.9 [9.03, 10.77]	28.2 [25.52, 30.94]	5.9 [5.43, 6.56]	16.9 [15.61, 18.27]
Female	6.9 [6.01, 7.71]	34.0 [30.19, 37.83]	4.8 [4.21, 5.35]	19.7 [17.95, 21.45]
**Marital Status**				
Currently married	8.6 [7.90, 9.32]	30.9 [28.31, 33.66]	5.5 [5.04, 5.97]	18.1 [16.79, 19.46]
Widowed	8.2 [6.80, 9.54]	29.0 [25.08, 32.95]	5.6 [4.59, 6.57]	17.5 [15.03, 19.86]
Others	9.8 [9.94, 13.63]	27.9 [20.90, 34.86]	4.1 [2.49, 5.68]	18.7 [10.69, 26.75]
**Educational attainment**				
Illiterate	9.9 [8.88, 10.96]	33.5 [29.69, 37.38]	6.0 [5.36, 6.69]	17.5 [16.42, 18.65]
Less than 5 years	10.5 [8.80, 12.16]	32.5 [28.83, 36.08]	6.6 [5.38, 7.73]	20.7 [17.73, 23.57]
5–9 years completed	9.1 [7.74, 10.41]	29.9 [26.49, 33.36]	5.6 [4.75, 6.41]	18.5 [16.82, 20.11]
10 years or more	4.3 [3.43, 5.19]	23.5 [16.35, 30.60]	3.3 [2.51, 4.03]	17.3 [11.3, 23.39]
**MPCE Quintile**				
Poorest	9.2 [7.80, 10.64]	30.2 [27.29, 33.05]	5.8 [5.00, 6.61]	16.9 [15.42, 18.34]
Poorer	9.2 [8.02, 10.45]	28.2 [25.45, 30.87]	5.1 [4.40, 5.75]	17.1 [15.57, 18.59]
Middle	9.0 [7.78, 10.27]	33.0 [30.20, 35.85]	5.6 [4.57, 6.57]	17.8 [16.08, 19.58]
Richer	8.3 [6.83, 9.67]	29.2 [25.83, 32.51]	5.9 [4.87, 6.92]	19.3 [17.30, 21.25]
Richest	6.7 [5.33, 8.07]	32.7 [24.02, 41.38]	4.9 [3.87, 5.8]	20.8 [14.01, 27.61]
**Residence**				
Rural	5.9 [4.94, 6.87]	29.9 [24.04, 35.77]	4.1 [3.37, 4.73]	19.2 [14.56, 23.87]
Urban	9.8 [9.04, 10.54]	30.8 [29.26, 32.42]	5.9 [5.44, 6.39]	17.7 [16.89, 18.58]
**Caste**				
Scheduled Tribes	8.9 [6.29, 11.62]	28.8 [24.62, 33.04]	5.6 [4.44, 6.79]	19.7 [17.54, 21.81]
Scheduled Castes	9.1 [7.88, 10.41]	32.7 [29.45, 35.96]	7.1 [6.03, 8.15]	17.0 [15.39, 18.65]
OBC	8.8 [7.89, 9.70]	31.6 [27.77, 35.45]	5.1 [4.52, 5.63]	17.7 [15.36, 20.08]
Others	7.6 [6.49, 8.65]	27.0 [24.44, 29.63]	4.6 [3.95, 5.33]	18.8 [16.74, 20.85]
**Religion**				
Hindu	8.5 [7.84, 9.17]	29.2 [27.62, 30.67]	5.5 [5.03, 5.89]	18.3 [16.89, 19.74]
Muslim	8.4 [6.44, 10.35]	39.5 [28.76, 50.30]	5.9 [4.51, 7.41]	15.9 [13.55, 18.38]
Christian	10.1 [7.03, 13.19]	22.2 [16.76, 27.71]	3.6 [2.53, 4.62]	10.6 [8.09, 13.01]
Others	8.9 [5.93, 11.99]	37.0 [29.76, 44.25]	6.2 [3.59, 8.84]	25.0 [20.05, 29.96]
**Smoke/Substance use**				
Yes	10.1 [9.07, 11.02]	33.0 [30.74, 35.31]	6.6 [5.91, 7.20]	19.1 [17.76, 20.45]
No	7.4 [6.64, 8.24]	28.9 [25.40, 32.43]	4.6 [4.05, 5.04]	17.2 [15.49, 18.93]
**Practicing Exercise**				
Yes	6.1 [4.47, 7.64]	32.5 [23.32, 41.75]	4.4 [3.34, 5.52]	17.0 [14.17, 19.85]
Rarely/ Never	8.9 [8.26, 9.56]	30.3 [28.12, 32.47]	5.6 [5.16, 6.00]	18.2 [16.84, 19.47]
**Health Insurance**				
No	8.5 [7.74, 9.16]	30.7 [28.29, 33.07]	5.3 [4.82, 5.72]	17.8 [16.84, 18.78]
Yes	8.9 [7.73, 10.01]	30.2 [25.70, 34.77]	6.2 [5.35, 7.05]	18.9 [14.93, 22.82]
**Region**				
North	8.8 [7.35, 10.17]	29.4 [26.43, 32.43]	5.0 [4.13, 5.91]	15.7 [13.75, 17.65]
Central	8.9 [7.19, 10.79]	30.2 [26.48, 33.86]	5.7 [4.35, 6.18]	15.4 [13.72, 17.04]
East	8.7 [7.33, 10.02]	24.6 [22.10, 26.99]	5.7 [4.86, 6.59]	14.5 [13.11, 15.79]
Northeast	6.4 [4.84, 7.98]	6.8 [4.92, 8.72]	4.3 [3.35, 5.29]	2.9 [1.98, 3.73]
West	8.9 [7.54, 10.31]	46.9 [43.44, 50.48]	6.8 [5.68, 7.82]	33.2 [30.78, 35.58]
South	8.1 [7.01, 9.12]	27.7 [21.89, 33.46]	4.8 [4.08, 5.54]	15.9 [11.69, 20.12]

Age was adjusted for sex; sex was adjusted for age and all other variables were adjusted for age and sex

Table 3 presents the age-sex adjusted estimates of ever stopped work and limiting work by type and number of chronic diseases. The prevalence of ever stopped work and limiting paid work due to chronic diseases was higher among those who had the chronic disease compared to who did not had across each of the eight diseases category. For instance, respondent who have been diagnosed with hypertension, 8.3% had ever stopped work compare to 6.4% who did not had hypertension. Similarly, among those with hypertension 30.6% had limiting work compared to 20.8% who did not had hypertension. The proportion of older adults who stopped work/ had limiting work was highest in case of stroke (21.1%, 95% CI: 15.29–28.26) and (51.6%, 95% CI: 40.82–62.16) respectively followed by neurological or psychiatric problems. Prevalence of both the outcome variables increased with the increase in the number of chronic diseases. For instance, the proportion of older adults who ever stopped work varies from 5.4% (95% CI: 4.98–5.88) among those with no chronic condition to 19.3% (95% CI: 10.25–33.22) among those with five or more chronic conditions. The pattern was similar in case of limiting paid work. A significant gap is found in the prevalence of stopped working and limiting work between the two groups of population, one who have been diagnosed with diabetes/hypertension and the other who have not.

**Table 3:** Proportion of middle-aged adults and elderly ever stopped work for 1 year or more and limiting paid work by type of chronic diseases in India, LASI 2017-18

	Ever stopped work for 1 year or more	Limiting paid work
	***N* = 1,660**	***N* = 2,973**
	**Prevalence 95% CI**	**Prevalence 95% CI**
**Hypertension**		
Yes	8.3 [7.31,9.36]	30.6 [26.76,34.65]
No	6.4 [6.01,6.82]	20.8 [19.67,21.87]
**Diabetes**		
Yes	8.5 [7.20,9.89]	35.0 [27.69,43.02]
No	6.7 [6.26,7.08]	21.5 [20.49,22.53]
**Cancer**		
Yes	11.4 [7.74,16.56]	40.3 [29.90,51.54]
No	6.8 [6.44,7.23]	22.6 [21.40,23.86]
**Chronic lung disease**		
Yes	10.1 [8.56,11.84]	38.1 [31.48,45.12]
No	6.6 [6.23,7.05]	21.9 [20.63,23.14]
**Chronic heart diseases**		
Yes	13.3 [9.99,17.55]	42.9 [36.98,49.11]
No	6.6 [6.25,7.02]	22.2 [20.95,23.44]
**Stroke**		
Yes	21.1 [15.29,28.26]	51.6 [40.82,62.16]
No	6.6 [6.20,6.95]	22.4 [21.16,23.62]
**Arthritis**		
Yes	9.0 [8.10,10.03]	34.0 [31.18,37.02]
No	6.5 [6.05,6.92]	21.1 [19.72,22.45]
**Neurological or psychiatric problems**		
Yes	18.3 [13.52,24.32]	36.5 [29.32,44.26]
No	6.6 [6.22,6.98]	22.4 [21.22,23.69]
**Number of Chronic diseases**		
0	5.4 [4.98,5.88]	18.0 [16.69,19.30]
1	7.9 [7.24,8.62]	26.4 [24.97,27.90]
2	8.8 [7.34,10.56]	36.1 [28.99,43.94]
3	13.0 [9.62,17.38]	50.5 [42.68,58.29]
4	20.3 [14.95,26.99]	50.8 [38.75,62.68]
5+	19.3 [10.25,33.22]	70.8 [44.78,87.92]

Table 4 shows result of propensity matching score of ever stopped work and limiting paid work. controlling for socio-demographic and economic covariates. The estimated ATT in treated and control groups are 0.085 and 0.046 respectively, suggesting that the population who had chronic condition, if they would not have, then 3.6% of them would not stop working. ATU result for controlled group indicates that among those individuals who had no chronic disease, if they would have chronic disease, then only 10.4% of them would stop working. ATE results indicate the average treatment effect and from the table, the difference in ATE is 4.8%. This indicates that after matching, the population with chronic disease are 4.8% more likely to stop working.

**Table 4:** Result of propensity matching score of ever stopped work or limiting work

Having any chronic disease vs. not having any chronic disease	Treated	Control	Differences	S.E.	T-test
**Ever stopped Work in 1 year or more**					
Unmatched	0.085	0.049	0.036	0.002	16.22
ATT	0.085	0.046	0.039	0.005	8.25
ATU	0.049	0.104	0.055	.	
ATE			0.048		
**Limiting Paid work**					
Unmatched	0.253	0.141	0.112	0.004	25.95
ATT	0.253	0.125	0.128	0.008	15.17
ATU	0.141	0.253	0.112	.	
ATE			0.118	.	

*ATT* Average treatment effect on the treated, *ATU* Average treatment effect on the untreated, *ATE* Average treatment effect

Similarly, the unmatched sample estimate for limiting paid work shows that individuals having any chronic disease are 11% more likely to have increased limiting paid work compared with the ones not having any chronic disease. The estimated ATT values in treated and control groups are 0.253 and 0.141 respectively, indicating that population who had chronic condition, if they would not have, then only 12.5% of them would limit paid work. ATU result for controlled group indicates that among those individuals who had no chronic disease, if they would have chronic disease, then only 25.3% of them would limit paid work. ATE results indicate the average treatment effect and from the table, the difference in ATE is 11.8%. this indicates that after matching, the population with chronic disease are 12% more likely to stop working.

The propensity score results for ever stopped work for 1 year or more and limiting paid work suggest that individual having any chronic disease is indeed associated with greater ever stopped work and limiting paid work.

Table 5 presents the odds ratio of ever stopped work using three regression models. In first model, we have included the number of chronic diseases while in model 2, the socio-demographic factors along with chronic diseases were included. In model 3, economic condition of the household, health insurance along with behavioural factors were included. Noticeably, the odds ratio of the number of chronic diseases show significant variation even after adjusting for socio-economic and demographic covariates. The odds of stopping work among those with 5 and more chronic disease were 4 times higher (OR: 4.17, 95% CI: 1.99–8.75) as compared to those having no chronic disease. Similarly, the odds of ever stopped work was significantly lower among females (OR: 0.70, 95% CI: 0.62–0.79) compared to males. By type of residence, the likelihood of ever stopped work was 1.6 times higher among rural residents (OR: 1.60 95%, CI 1.34–1.90) compared to urban residents. For all other demographic variables except the number of chronic diseases, the pattern remains similar to that of model 2. However, the odds of stopping work were 1.13 (OR: 1.13, 95% CI: 0.95–1.34) times higher among richer compared to that of poorer. The odds of stopping work declined with each gradient of educational level. Those who were using any substance, the odds of stopping work was 1.26 times higher (OR: 1.26, 95% CI: 1.12–1.43) compare to those who don’t. Similarly, among those who do not practice exercise or practices rarely, the odds of stopping work was 1.15 times higher (OR: 1.15, 95% CI: 0.94–1.40) than those who practices exercise.

**Table 5:** Adjusted odds ratio for ever stopped wok by socioeconomic and demographic characteristics among middle aged and elderly people in India, 2017-18

	Unadjusted		Adjusted			
	**Model 1**		**Model 2**		**Model 3**	
	**OR**	**95% CI**	**OR**	**95% CI**	**OR**	**95% CI**
**Number of Chronic diseases**						
0 ®						
1	1.50*	1.32–1.71	1.56*	1.37–1.77	1.61*	1.42–1.83
2	1.69*	1.36–2.10	1.69*	1.41–2.03	1.86*	1.56–2.22
3	2.62*	1.84–3.72	2.52*	1.98–3.21	2.69*	2.12–3.42
4	4.46*	3.05–6.53	4.56*	3.05–6.81	4.72*	3.14–7.10
5+	4.17*	1.99–8.75	4.88*	2.29–10.41	5.70*	2.61–12.44
**Age**						
75+ ®						
< 45			1.24*	0.78–1.96	1.12*	0.78–1.62
45–54			1.07*	0.87–1.33	1.14*	0.92–1.42
55–64			1.10*	0.89–1.35	1.13*	0.91–1.39
65–74			1.05*	0.85–1.29	1.06*	0.86–1.30
**Sex**						
Male ®						
Female			0.70*	0.62–0.79	0.70*	0.60–0.80
**Residence**						
Urban®						
Rural			1.60*	1.34–1.90	1.44*	1.24–1.67
**Caste**						
** Others**®						
Scheduled Tribes			1.47*	1.22–1.77	1.23*	1.03–1.46
Scheduled Castes			1.19*	0.95–1.49	1.04*	0.83–1.32
OBC			1.15*	0.99–1.32	1.07*	0.93–1.24
**Religion**						
Muslim®						
Hindu			0.84*	0.69–1.01	0.92*	0.75–1.12
Christian			0.75*	0.54–1.05	0.89*	0.64–1.24
Others			0.82*	0.57–1.18	1.00*	0.69–1.46
**Marital Status**						
** Others**®						
Currently married			1.01*	0.74–1.38	0.99*	0.72–1.35
Widowed			0.98*	0.70–1.36	0.94*	0.67–1.31
**Region**						
North®						
Central			0.96*	0.79–1.17	1.03*	0.84–1.26
East			0.96*	0.80–1.15	0.97*	0.81–1.17
Northeast			0.76*	0.60–0.95	0.72*	0.57–0.91
West			1.20*	1.00-1.43	1.23*	1.02–1.47
South			0.96*	0.79–1.17	0.93*	0.77–1.11
**MPCE Quintile**						
Poorer®						
Poorest					1.03*	0.89–1.20
Middle					1.08*	0.92–1.27
Richer					1.13*	0.95–1.34
Richest					1.09*	0.90–1.32
**Educational attainment**						
Illiterate®						
Less than 5 years					1.06*	0.90–1.24
5–9 years completed					0.96*	0.82–1.11
10 years or more					0.55*	0.44–0.69
**Smoke/Substance use**						
** No**®						
Yes					1.26*	1.12–1.43
**Practicing Exercise**						
Yes®						
Rarely/ Never					1.15*	0.94–1.40
**Health Insurance**						
No®						
Yes					1.16*	1.02–1.31

® indicates reference category

* *p* < 0.05, values in the parentheses are 95% confidence interval

Table 6 shows the unadjusted and adjusted odds ratio for limiting paid work. The odds of the number of chronic diseases show significant variation even after adjusting for socio-economic and demographic covariates. For instance, compared to those having no chronic disease, person with 2 chronic diseases were significantly more likely to have limiting paid work (OR: 2.58, 95% CI: 1.84–3.62). The likelihood of limiting work was significantly higher among females (OR: 1.17, 95% CI: 1.00-1.36) and those residing in rural areas (OR: 1.08, 95% CI: 0.86–1.34) as compared to that of males. Similarly, the odds of limiting paid work was higher among ST (OR: 1.31, 95% CI: 1.10–1.55) followed by SC (OR: 1.34, 95% CI: 1.10–1.63) compared to the other caste. For all other demographic variables in model 3, the pattern remains similar that to of model 2 however for MPCE quintile the chances of limiting paid work was 1.45 times higher among richest quintile (OR: 1.45, 95% CI: 1.11–1.90) compare to that of poorer.

**Table 6:** Adjusted odds ratio for limiting paid work by socioeconomic and demographic characteristics among middle aged and elderly people in India, 2017-18

	Unadjusted		Adjusted			
	**Model 1**		**Model 2**		**Model 3**	
	**OR**	**95% CI**	**OR**	**95% CI**	**OR**	**95% CI**
**Number of Chronic diseases**						
0®						
1	1.64*	1.46–1.84	1.62*	1.43–1.83	1.64*	1.45–1.86
2	2.58*	1.84–3.62	2.50*	1.82–3.45	2.66*	1.97–3.59
3	4.66*	3.36–6.47	3.83*	2.80–5.25	3.88*	2.82–5.33
4	4.71*	2.87–7.73	5.04*	3.09–8.23	5.17*	3.15–8.48
5+	11.09*	3.69–33.35	10.27*	2.54–41.46	11.94*	2.68–53.19
**Age**						
75+®						
< 45			0.52*	0.29–0.92	0.52*	0.30–0.93
45–54			0.43*	0.30–0.61	0.44*	0.30–0.65
55–64			0.50*	0.35–0.70	0.51*	0.36–0.73
65–74			0.78*	0.55–1.09	0.78*	0.55–1.11
**Sex**						
Male®						
Female			1.17*	1.00-1.36	1.16*	0.92–1.47
**Residence**						
Urban®						
Rural			1.08*	0.86–1.34	1.03*	0.86–1.22
**Caste**						
Others®						
Scheduled Tribes			1.31*	1.10–1.55	1.22*	1.01–1.46
Scheduled Castes			1.34*	1.10–1.63	1.26*	1.03–1.55
OBC			1.24*	1.05–1.45	1.21*	1.02–1.44
**Religion**						
Muslim®						
Hindu			0.74*	0.51–1.06	0.79*	0.56–1.12
Christian			0.61*	0.38–0.96	0.65*	0.41–1.02
Others			0.92*	0.61–1.38	1.04*	0.70–1.56
**Marital Status**						
Others®						
Currently married			0.94*	0.62–1.43	0.94*	0.63–1.42
Widowed			0.91*	0.59–1.40	0.89*	0.58–1.37
**Region**						
North®						
Central			0.98*	0.84–1.14	1.05*	0.89–1.22
East			0.82*	0.72–0.94	0.85*	0.74–0.98
Northeast			0.16*	0.13–0.21	0.16*	0.12–0.21
West			2.48*	2.15–2.86	2.63*	2.28–3.03
South			0.94*	0.76–1.18	0.94*	0.77–1.14
**MPCE Quintile**						
Poorer®						
Poorest					1.06*	0.94–1.20
Middle					1.15*	1.01–1.31
Richer					1.14*	0.98–1.33
Richest					1.45*	1.11–1.90
**Educational attainment**						
Illiterate®						
Less than 5 years					0.97*	0.81–1.15
5–9 years completed					0.89*	0.76–1.05
10 years or more					0.69*	0.49–0.99
**Smoke/Substance use**						
No®						
Yes					1.20*	1.06–1.36
**Practicing Exercise**						
Yes®						
Rarely/ Never					0.95 .+++*	0.69–1.29
**Health Insurance**						
No®						
Yes					1.20*	1.00-1.45

® indicates reference category

* *p* < 0.05, values in the parentheses are 95% confidence interval

Additional file 1: Appendix 2 presents the estimated proportion of ever stopped work and limiting work among working age population (under 65) and 65 + by chronic diseases. In each of the variable, the proportion who stopped work was higher among those with any chronic disease compared to those without chronic diseases. The proportion of ever stopped worked for each of the diseases were higher among those in working age group compared to elderly (65+). However, the proportion of limiting work was higher for those 65+, in most of the chronic diseases.

## Discussion

This is the first ever population-based study that estimated the prevalence of ever stopped work and limiting paid work among middle aged and elderly in India. The key strength of our study is the use of the first and latest data from a high-quality, nationally representative, population-based ageing survey in India. Our study included sample of the middle-aged population, as well as the elderly population who have ever worked. This study fills the critical gaps in knowledge by investigating pattern and prevalence of limiting paid work and productivity loss among middle-aged and elderly in India and their association with chronic diseases and the validity of these findings has been confirmed by employing the robustness checks.

The results of age-sex adjusted estimates of ever stopped work and limiting work suggest that 7% of older adults ever stopped working and 23% had limiting work due to health-related issues. The prevalence of ever-stopped working and limiting work due to ill health is higher among those with a chronic condition compared to those who do not have that across socio-economic characteristics. As expected, the prevalence of ever-stopped work and limiting paid work are higher among the people who have even a single disease than who doesn’t and positively associated with age. The results of propensity score matching show that the difference in ATE is 4.8% and 12% which indicates after matching, the population with chronic disease are 4.8% and 12% more likely to stop working. Moreover, the prevalence of ever stopped work was higher among those in working age group compared to elderly (65+). However, the probability of limiting paid work was higher among elderly compared to working age group. Controlling for socio-demographic and economic factors, the probability for ever stopped work was lower among females but higher among rural dwellers. The probability of limiting paid work was higher among females, rural dwellers and people who had health insurance, also this was high among people belonging to comparatively higher MPCE groups. These findings are consistent with literature from low- and middle-income countries [35]. Second, we found educational attainment as significant predictors of ever stopped work and limiting paid work. In the case of full model (model 3) a significant decrease in stopping work and limiting paid work was observed with higher level of education. Zimmerman et al. addressed this and investigated that, those adults with relatively higher educational level are expected to have greater socio-economic resources to attain a healthy lifestyle, also they are well equipped with the health literacy level required to avail later in their lives [36].

We found each of the chronic disease are significantly associated with stopping work and limiting paid work. Overall, among the eight chronic health conditions, the chronic diseases with the strongest association to stopping work or limiting paid work were stroke followed by Neurological or psychiatric problems. Many stroke survivors experience poststroke spasticity resulting in inability to perform daily activities, further necessitating their management and treatment. This exerts a considerable economic burden due to treatment cost and lost productive days [37]. Results from a study also indicate that inability to complete neuropsychological tests at one-year post-injury is associated with non-productive activity [38]. The chance of ever stopped work by each of the chronic diseases was higher among adults in the prime working age group suggesting that chronic diseases significantly inhibit the work. Even after adjusting for other socioeconomic and demographic characteristics, number of chronic diseases is found to be important contextual unit for ever-stopped work and limiting paid work.

As per World global health (WHO) fact on non-communicable diseases 2021 showed that, 71% of all deaths caused by non-communicable each year 15 million people in age group 30 to 69 dies due to NCDs and 85% of them belong to low- and middle-income countries and 77% of all NCDs death takes place in low- and middle-income countries. Chronic disease does not only hinder individual productivity and wellbeing but also it brings economic and human working hours capital loss for the nation. The increased burden of chronic diseases among working population in low-income and middle-income countries that have inadequate health systems might increase the productivity loss and global inequality and instability.

Occurrence of chronic diseases among the working age group is expected to increase along with increasing share of elderly population in India [39]. Chronic disease poses greater risk of high medical expenditure and productivity loss at work for the working population. Our study reflects the very same notion. Evidences from this study on chronic diseases and productivity loss in India is new and staggering, with a demand of policy attention. At present, there is no official programme focusing on work place and chronic diseases in India. The first step in this direction is to create awareness followed by screening for growing non-communicable diseases, at least for employee working in public and private sectors to optimise the productivity potential. The burden of ill-health in terms of productivity loss will further increase if no programs are implemented to manage, control, or prevent chronic diseases among working middle-aged and elderly population in India. There need to be an investment in carefully designing workplace intervention by the policymakers and employers at population and individual level to turn away the adverse economic and health consequences of chronic diseases.

We acknowledge the following limitations of this study. First, the chronic diseases we used are self-reported and medically diagnosed. We believe that a higher proportion of population with chronic diseases has not remain undiagnosed. Second, we did not analysed by actual loss of wage / income due to lack of data. Despite these limitations, we believe that the findings serves as the first population based study on estimates of loss of productivity due to chronic diseases in India.

## Conclusion

This study has demonstrated that stopping work and limiting paid work were significantly associated with chronic diseases. The chronic diseases have their greatest impact on performance domain of productivity or limiting paid work. It could be used as an indicator of the performance of workplace health interventions and guide employers and policy makers towards better adjustments for employees with chronic diseases.

## Supplementary Information

**Additional file 1: Appendix 1.** Shows the questions asked to generate the two outcome variables. **Appendix 2.** Estimates of ever stopped work for 1 year or more and limiting paid work by types of chronic diseases, socioeconomic and demographic characteristics among elderly  and non-elderly in India, 2017-18.

## Data Availability

The datasets generated and/or analysed during the current study are available with the International Institute for Population Sciences, Mumbai, India repository and could be accessed from the following link: https://iipsindia.ac.in/sites/default/files/LASI_DataRequestForm_0.pdf. Those who wish to download the data have to follow the above link. This link leads to a data request form designed by International Institute for Population Sciences. After completing the form, it should be mailed to: datacenter@iips.net for further processing. After successfully sending the mail, individual will receive the data in a reasonable time.
