# Supplementary material for: Chronic diseases and productivity loss among middle-aged and elderly in India
Source: BMC Public Health. 2022 Dec 16;22:2356. doi: 10.1186/s12889-022-14813-2 (PMC9756765; doi:10.1186/s12889-022-14813-2)
Supplement: Supplementary file 1 — Additional file 1: Appendix 1. Shows the questions asked to generate the two outcome variables. Appendix 2. Estimates of ever stopped work for 1 year or more and limiting paid work by types of chronic diseases, socioeconomic and demographic characteristics among elderly and non-elderly in India, 2017-18. [file 12889_2022_14813_MOESM1_ESM.docx]

Appendix 1. shows the questions asked to generate the two outcome variables.

Appendix 1

| **Appendix 1. Questions used to generate the outcome variables** | | |
| --- | --- | --- |
| Variables | Source of measurement | Measurement |
| Dependent variables: | | |
| 1) Stopping work due to health reasons | |  |
| Ever stopped working | WE007: [Ask only if ever worked]Have you ever stopped working for one year or more at a time due to reasons of family, health, education, economic recession, natural disasters, etc.? | 1. Yes |
|  |  | 2. No |
|  |  |  |
| Reason for stopping work | WE008: [Ask only if WE007=1] What was the main reason for this interruption? (Only considered WE008=3 for analysis) | 1. Marriage |
|  |  | 2. Childcare |
|  |  | 3. Health problems |
|  |  | 4.Education |
|  |  | 5. Other family related reason |
|  |  | 6. Layoff/closure of work unit |
|  |  | 7. Natural disaster |
|  |  | 8. Others |
| 2) Limiting paid work | HT300: [Ask only if ever worked or currently not working and temporarily laid off] Now I want to ask how your health affects paid work activities. Do you have any | 1. Yes 2. No |
|  | impairment or health problem that limits the kind or amount of paid work you can do? |  |

Appendix 2. Estimates of ever stopped work for 1 year or more and limiting paid work by types of chronic diseases, socioeconomic and demographic characteristics among elderly and non-elderly in India, 2017-18

Appendix 2

| **Appendix 1. Estimates of ever stopped work for 1 year or more and limiting paid work by types of chronic diseases, socioeconomic and demographic characteristics among elderly and non-elderly in India, 2017-18** | | | | | | | | | |
| --- | --- | --- | --- | --- | --- | --- | --- | --- | --- |
|  | **Ever stopped working due to health** | | | |  | **Limiting paid work** | | | |
|  | **<65 age group** | | **>65 age group** | |  | **<65 age group** | | **>65 age group** | |
|  | **Prevalence** | **95% CI** | **Prevalence** | **95% CI** |  | **Prevalence** | **95% CI** | **Prevalence** | **95% CI** |
| **Hypertension** |  |  |  |  |  |  |  |  |  |
| 1 Yes | 8.7 | [7.26,10.34] | 7.7 | [6.62,8.96] |  | 28.4 | [23.53,33.90] | 37.4 | [33.98,40.92] |
| 2 No | 6.2 | [5.76,6.77] | 6.8 | [6.13,7.45] |  | 18.5 | [17.32,19.79] | 31.0 | [28.66,33.52] |
|  |  |  |  |  |  |  |  |  |  |
| **Diabetes** |  |  |  |  |  |  |  |  |  |
| 1 Yes | 9.0 | [7.34,11.11] | 7.5 | [5.84,9.61] |  | 32.0 | [22.82,42.79] | 45.8 | [40.30,51.38] |
| 2 No | 6.5 | [6.00,7.07] | 7.0 | [6.40,7.61] |  | 19.3 | [18.14,20.46] | 31.1 | [29.03,33.32] |
|  |  |  |  |  |  |  |  |  |  |
| **Cancer** |  |  |  |  |  |  |  |  |  |
| 1 Yes | 12.7 | [7.95,19.66] | 9.5 | [4.63,18.44] |  | 37.0 | [25.99,49.65] | 54.9 | [31.06,76.64] |
| 2 No | 6.7 | [6.23,7.25] | 7.0 | [6.47,7.64] |  | 20.3 | [18.86,21.79] | 32.5 | [30.53,34.57] |
|  |  |  |  |  |  |  |  |  |  |
| **Chronic lung disease** |  |  |  |  |  |  |  |  |  |
| 1 Yes | 11.1 | [8.73,14.11] | 8.8 | [7.17,10.88] |  | 36.3 | [28.00,45.52] | 43.0 | [36.96,49.19] |
| 2 No | 6.5 | [6.01,7.06] | 6.9 | [6.28,7.50] |  | 19.6 | [18.14,21.12] | 31.8 | [29.74,33.98] |
|  |  |  |  |  |  |  |  |  |  |
| **Chronic heart diseases** |  |  |  |  |  |  |  |  |  |
| 1 Yes | 17.5 | [11.93,24.96] | 9.0 | [6.37,12.46] |  | 40.0 | [32.44,48.10] | 50.3 | [41.62,59.01] |
| 2 No | 6.5 | [6.00,6.98] | 6.9 | [6.38,7.56] |  | 19.9 | [18.48,21.44] | 31.9 | [29.90,34.03] |
|  |  |  |  |  |  |  |  |  |  |
| **Stroke** |  |  |  |  |  |  |  |  |  |
| 1 Yes | 25.3 | [15.01,39.48] | 17.3 | [13.26,22.34] |  | 52.7 | [38.02,66.99] | 49.2 | [37.87,60.61] |
| 2 No | 6.5 | [6.04,7.00] | 6.7 | [6.15,7.31] |  | 20.1 | [18.65,21.56] | 32.3 | [30.28,34.36] |
|  |  |  |  |  |  |  |  |  |  |
| **Arthritis** |  |  |  |  |  |  |  |  |  |
| 1 Yes | 8.9 | [7.67,10.22] | 9.2 | [7.88,10.83] |  | 29.2 | [25.96,32.61] | 47.8 | [43.46,52.07] |
| 2 No | 6.4 | [5.91,7.03] | 6.5 | [5.92,7.18] |  | 19.2 | [17.66,20.89] | 29.4 | [27.22,31.72] |
|  |  |  |  |  |  |  |  |  |  |
| **Neurological or psychiatric problems** |  |  |  |  |  |  |  |  |  |
| 1 Yes | 22.5 | [15.61,31.24] | 11.5 | [7.32,17.67] |  | 35.5 | [26.90,45.26] | 39.5 | [28.95,51.11] |
| 2 No | 6.4 | [5.96,6.93] | 6.9 | [6.37,7.53] |  | 20.1 | [18.67,21.61] | 32.5 | [30.45,34.54] |
|  |  |  |  |  |  |  |  |  |  |
| **Number of chronic diseases** |  |  |  |  |  |  |  |  |  |
| 0 | 5.3 | [4.83,5.95] | 5.6 | [4.88,6.28] |  | 16.3 | [14.84,17.75] | 25.9 | [23.77,29.83] |
| 1 | 7.9 | [7.00,8.75] | 7.9 | [6.95,9.25] |  | 23.7 | [22.27,25.54] | 36.7 | [32.30,38.69] |
| 2 | 9.0 | [6.86,11.80] | 8.4 | [6.88,10.51] |  | 34.0 | [24.80,44.51] | 42.2 | [37.97,47.95] |
| 3 | 15.1 | [10.71,24.78] | 9.7 | [6.99,12.36] |  | 43.6 | [36.81,58.97] | 55.1 | [47.51,64.40] |
| 4 | 22.8 | [22.45,45.99] | 10.4 | [8.12,19.39] |  | 39.4 | [27.76,57.22] | 62.9 | [51.73,83.59] |
| 5+ | 40.4 | [17.46,55.42] | 12.1 | [2.67,25.39] |  | 70.4 | [56.63,94.47] | 68.4 | [16.58,84.92] |
|  |  |  |  |  |  |  |  |  |  |
| **MPCE Quintile** |  |  |  |  |  |  |  |  |  |
| 1 | 6.9 | [6.03,7.86] | 7.0 | [5.96,8.15] |  | 18.7 | [17.29,20.10] | 30.9 | [27.54,34.37] |
| 2 | 6.2 | [5.49,6.96] | 7.7 | [6.63,8.96] |  | 17.9 | [16.50,19.32] | 33.0 | [29.50,36.68] |
| 3 | 6.9 | [6.00,7.90] | 7.1 | [5.92,8.57] |  | 20.6 | [19.10,22.22] | 34.0 | [29.89,38.44] |
| 4 | 7.6 | [6.29,9.11] | 6.5 | [5.08,8.39] |  | 21.0 | [18.88,23.27] | 34.0 | [28.13,40.30] |
| 5 | 5.3 | [4.40,6.40] | 7.2 | [5.75,8.87] |  | 23.9 | [17.41,31.80] | 28.1 | [23.88,32.75] |
|  |  |  |  |  |  |  |  |  |  |
| **Education** |  |  |  |  |  |  |  |  |  |
| Illiterate | 7.5 | [6.60,8.46] | 7.1 | [6.38,7.87] |  | 22.3 | [20.36,24.29] | 32.2 | [29.67,34.91] |
| Less_than5years | 8.3 | [7.17,9.53] | 8.7 | [7.11,10.70] |  | 22.0 | [19.57,24.62] | 35.4 | [30.63,40.48] |
| 5-9yearscompleted | 7.1 | [6.32,7.96] | 8.4 | [6.86,10.33] |  | 19.3 | [17.77,21.03] | 35.2 | [31.31,39.21] |
| 10yearsormore | 4.0 | [3.36,4.85] | 3.8 | [2.80,5.07] |  | 16.7 | [12.16,22.41] | 27.3 | [19.67,36.59] |
|  |  |  |  |  |  |  |  |  |  |
| **Residence** |  |  |  |  |  |  |  |  |  |
| 1 | 7.2 | [6.73,7.71] | 7.6 | [6.92,8.31] |  | 19.8 | [19.07,20.63] | 31.5 | [29.56,33.59] |
| 2 | 5.1 | [4.17,6.11] | 5.7 | [4.73,6.94] |  | 21.1 | [16.65,26.38] | 35.4 | [30.24,41.00] |
|  |  |  |  |  |  |  |  |  |  |
| **Caste** |  |  |  |  |  |  |  |  |  |
| SC | 8.4 | [7.13,9.82] | 7.7 | [6.45,9.06] |  | 20.9 | [19.00,22.95] | 30.8 | [26.93,34.90] |
| ST | 6.3 | [5.19,7.74] | 6.3 | [4.47,8.80] |  | 19.7 | [17.83,21.67] | 31.0 | [25.46,37.14] |
| OBC | 6.6 | [5.82,7.45] | 7.2 | [6.37,8.07] |  | 20.5 | [17.78,23.53] | 36.0 | [32.82,39.28] |
| Others | 5.8 | [5.18,6.58] | 6.5 | [5.37,7.75] |  | 19.9 | [18.30,21.69] | 28.2 | [24.78,32.00] |
|  |  |  |  |  |  |  |  |  |  |
| **Religion** |  |  |  |  |  |  |  |  |  |
| Hindu | 6.7 | [6.17,7.25] | 6.9 | [6.27,7.54] |  | 19.8 | [18.55,21.08] | 32.6 | [30.41,34.94] |
| Muslim | 6.9 | [5.57,8.55] | 7.6 | [5.85,9.93] |  | 25.5 | [17.51,35.59] | 31.0 | [25.80,36.75] |
| Christian | 7.7 | [3.76,15.01] | 7.4 | [5.06,10.70] |  | 13.4 | [8.27,20.87] | 30.9 | [23.09,40.03] |
| Others | 7.3 | [5.09,10.32] | 8.0 | [5.38,11.82] |  | 26.8 | [22.64,31.48] | 39.5 | [29.95,49.91] |
|  |  |  |  |  |  |  |  |  |  |
| **Marital Status** |  |  |  |  |  |  |  |  |  |
| Currently married | 6.8 | [6.27,7.43] | 7.6 | [6.89,8.45] |  | 20.2 | [18.66,21.90] | 32.6 | [30.14,35.16] |
| Widowed | 6.7 | [5.69,7.82] | 5.8 | [5.01,6.79] |  | 21.8 | [19.16,24.79] | 32.7 | [29.36,36.26] |
| Others | 5.0 | [3.56,6.89] | 9.2 | [5.63,14.70] |  | 19.7 | [13.69,27.42] | 32.6 | [22.70,44.30] |
|  |  |  |  |  |  |  |  |  |  |
| **Smoke/Substance abuse** |  |  |  |  |  |  |  |  |  |
| 1 Yes | 8.8 | [7.88,9.89] | 8.2 | [7.39,9.17] |  | 20.9 | [19.67,22.24] | 32.5 | [30.17,35.00] |
| 2 No | 5.3 | [4.85,5.87] | 6.0 | [5.26,6.78] |  | 19.9 | [17.66,22.35] | 32.8 | [29.54,36.15] |
|  |  |  |  |  |  |  |  |  |  |
| **Practicing Exercise** |  |  |  |  |  |  |  |  |  |
| Yes | 5.6 | [4.49,6.98] | 4.8 | [3.54,6.47] |  | 20.7 | [14.44,28.69] | 39.6 | [30.29,49.72] |
| Rarely/Never | 6.8 | [6.32,7.35] | 7.3 | [6.72,7.98] |  | 20.3 | [18.96,21.77] | 31.8 | [29.88,33.85] |
|  |  |  |  |  |  |  |  |  |  |
| **Health Insurance** |  |  |  |  |  |  |  |  |  |
| No | 6.7 | [6.07,7.33] | 6.8 | [6.21,7.53] |  | 20.2 | [18.89,21.60] | 31.0 | [29.05,33.10] |
| Yes | 7.0 | [6.22,7.86] | 8.1 | [6.91,9.44] |  | 20.7 | [16.86,25.23] | 38.5 | [33.16,44.11] |
